# Molecular dissection of the migrating posterior lateral line primordium during early development in zebrafish

**DOI:** 10.1186/1471-213X-10-120

**Published:** 2010-12-13

**Authors:** Viviana E Gallardo, Jin Liang, Martine Behra, Abdel Elkahloun, Eduardo J Villablanca, Vincenzo Russo, Miguel L Allende, Shawn M Burgess

**Affiliations:** 1 Center for Genome Regulation. Facultad de Ciencias, Universidad de Chile. Casilla 653. Santiago, Chile; 2 National Human Genome Research Institute, National Institutes of Health, Bethesda, MD 20892, USA; 3 Department of Anatomy & Neurobiology, School of Medicine, Medical Sciences Campus, University of Puerto Rico, San Juan, PR 00936, USA; 4 Cancer Gene Therapy Unit, Cancer Immunotherapy and Gene Therapy Program, Scientific Institute H. San Raffaele, via Olgettina 58, 20132, Milan, Italy

## Abstract

**Background:**

Development of the posterior lateral line (PLL) system in zebrafish involves cell migration, proliferation and differentiation of mechanosensory cells. The PLL forms when cranial placodal cells delaminate and become a coherent, migratory primordium that traverses the length of the fish to form this sensory system. As it migrates, the primordium deposits groups of cells called neuromasts, the specialized organs that contain the mechanosensory hair cells. Therefore the primordium provides both a model for studying collective directional cell migration and the differentiation of sensory cells from multipotent progenitor cells.

**Results:**

Through the combined use of transgenic fish, Fluorescence Activated Cell Sorting and microarray analysis we identified a repertoire of key genes expressed in the migrating primordium and in differentiated neuromasts. We validated the specific expression in the primordium of a subset of the identified sequences by quantitative RT-PCR, and by *in situ *hybridization. We also show that interfering with the function of two genes, *f11r *and *cd9b*, defects in primordium migration are induced. Finally, pathway construction revealed functional relationships among the genes enriched in the migrating cell population.

**Conclusions:**

Our results demonstrate that this is a robust approach to globally analyze tissue-specific expression and we predict that many of the genes identified in this study will show critical functions in developmental events involving collective cell migration and possibly in pathological situations such as tumor metastasis.

## Background

The formation of an embryo and its organ systems requires the coordination of diverse cellular behaviors to achieve proper development of form and function. Cells must migrate, often collectively, and proliferate in a regulated way, while simultaneously carrying out specific developmental programs. A full characterization of these events requires a description of the cellular histories (lineage) and knowledge about the molecules that regulate these processes.

Active cell movements take place not only during the development of organisms, but also in processes that occur during adult life. For example, in the immune system, an effective immune response depends on the regulated traffic of its cellular components. Disrupted cell migration also contributes to several important pathological processes, including cancer and chronic inflammatory diseases such as rheumatoid arthritis and multiple sclerosis.

To date, most knowledge about cell migration is based on *in vitro *studies of single cells in two-dimensional cultures. These studies have allowed great progress on the intracellular events that take place during cell motility, elucidating the details of the cellular machinery driving migration. However, *in vivo *models of collective cell migration have been less intensely studied because of the inherent difficulty in undertaking such analyses. Recently, the migrating primordium of the zebrafish posterior lateral line has emerged as an attractive system for genetic analysis of cell migration and tissue organization and for understanding how these processes are controlled [1-9].

The lateral line is a mechanosensory system present in fish and amphibians that responds to water movements and is involved in a large variety of behaviors such as predator avoidance, prey detection, or swimming in schools [10,11]. This sensory system is formed by a number of discrete sense organs, the neuromasts, distributed over the body in species-specific patterns. The neuromasts of the head form the anterior lateral line (ALL), while the neuromasts of the trunk and tail, including those on the caudal fin, form the posterior lateral line (PLL). In zebrafish, the embryonic PLL comprises 7-8 neuromasts and its development begins at 20 hours post-fertilization (hpf) when a group of about 120 cranial placodal cells delaminates and begins to migrate collectively along the horizontal myoseptum towards the tail of the developing larva [12]. During its journey, the PLL primordium deposits groups of 15-20 cells (proneuromasts) at regular intervals and, a few hours after deposition, each proneuromast differentiates into a functional neuromast. They contain at least three cell types, including the mechanosensory hair cells and, therefore, the primordium contains multipotent progenitors for different cell types. Progenitors are also set aside within mature neuromasts, as damage to these organs is followed by functional recovery due to robust regeneration of hair cells and other structural elements [13-19].

One of the essential features of the migrating lateral line primordium is that its cells must become organized in order for them to migrate coherently and produce a functional organ. Today, it is known that the primordium of the LLP contains mesenchymal-like cells at its leading edge and, towards the trailing edge, 2 to 3 groups of rosette-shaped polarized cell clusters are formed, each corresponding to a proneuromast. Moreover, recent studies have shown that ligands of the FGF family, *fgf3 *and *fgf10*, are essential for the internal organization, patterning and migration of the primordium and therefore for correct neuromast deposition [4,5]. These ligands, together with Wnt pathway activation, are responsible for the restricted expression of the chemokine receptors CXCR4b and CXCR7b in the different compartments of the primordium [6]. Both these chemokine receptors, and the SDF1a/CCL12 ligand, are critical for proper directional guidance of the primordium cells as inactivation of these molecules produces alterations in the migration pattern of the primordium [1-3,20,21]. While directionality of migration is affected under these conditions, the migratory behavior of the cells themselves is not impaired, suggesting that independent pathways may regulate directionality and movement of the cells. Thus, there are likely to be numerous additional molecules involved in the process.

As the PLL primordium migrates, cells are simultaneously dividing and undergoing developmental programs that will build the functional lateral line. Hair cells and accessory cells derive from migratory primordium cells and their fates become apparent prior to neuromast deposition. The proneural master control gene for hair cell fate, *atonal homolog 1a *(*atoh1a*), is expressed in the center of the rosettes that form successively within the primordium [22,23]. Likewise, the neurogenic genes *delta *and *Notch *participate in cell type specification in neuromasts and are detected in presumptive neural and accessory cell progenitors, respectively [22,24].

The identification of new molecules that control the directional movement of the primordium, cell proliferation and subsequent differentiation of hair cells and other cell types will contribute to a better understanding of the processes leading to development of the mechanosensory lateral line, which shares many physiological properties with the mammalian auditory system. More importantly, formation of this organ shares molecular mechanisms with processes involved in cancer metastasis [8] and immune system cell migration [25].

In this study, we carried out a global survey of genes expressed in the PLL primordium and in maturing neuromasts. We have taken advantage of the ClaudinB:GFP transgenic zebrafish line (*cldnb:gfp*) [1], in which primordium and neuromast cells specifically express the membrane-bound enhanced Green Fluorescent Protein (eGFP). We have isolated GFP expressing cells as well as non-labeled cells at two different developmental stages (36 and 48 hpf) using Fluorescence Activated Cell Sorting (FACS). We show that comparison of expression profiles from GFP-positive and GFP-negative cells by microarray analysis allows identification of genes highly expressed in the migrating primordium (36 hpf set) and in deposited neuromasts (48 hpf set). Thus, the expression profiles of lateral line precursors during and after the migratory phase could be compared. We further concentrated on the 36 hpf set of data, to find molecules belonging to diverse cellular and developmental processes. Using this approach, we demonstrated its utility for the identification of genes involved in morphogenesis, migration and cell type specification within the migrating PLL primordium in the zebrafish. Importantly, some of the identified gene products can also open new insights into a more global comprehension of cell migration, which may be crucial to create efficient therapies to treat human diseases such as cancer and autoimmune disease.

## Results

### Isolation of primordium cells (36 hpf set)

**Figure 1:**
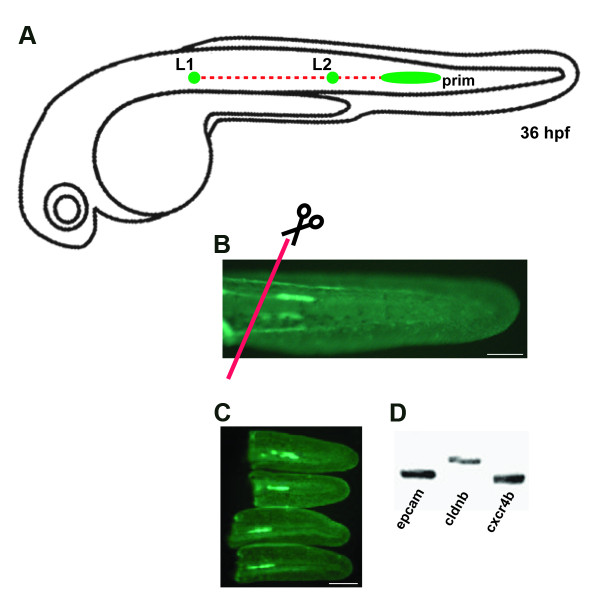
**Section of the transgenic line *cldnb:gfp***. (A) Schematic drawings of a migrating primordium of the posterior lateral line along the horizontal myoseptum at 36 hpf. (B) Transgenic *cldnb:gfp *embryo at 36 hpf, when tails were sectioned (red line). (C) Sectioned tails with GFP primordium. (d) RT-PCR of RNA derived from dissected tails of 36 hpf embryos with primer specific to three genes known to be expressed in the primordium at this stage. L1: neuromast 1, L2: neuromast 2, prim: LLP primordium. Scale bars are 50 μm in (b-c).

**Figure 2:**
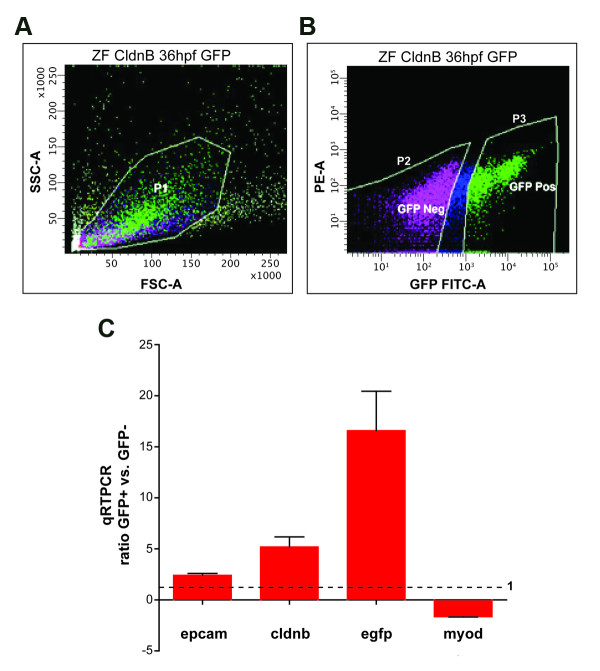
**Fluorescence activated cell sorting (FACS) of dissociated *cldnb:gfp *tails**. (A) Plot showing the P1 gate used to sort cells from tails of transgenic (*cldnb:gfp*) embryos, according to cell size (forward scatter) vs. granularity (side scatter). (B) Plot showing GFP fluorescence intensity of cells vs. Phycoerythrin (PE-A). P2 and P3 demarcate cells sorted as GFP- and GPP+, respectively. (C) Quantitative RT-PCR analysis (qRT-PCR) was performed of RNA derived from GFP+ and GFP- cells from tails of 36 hpf embryos. Real time PCR ratio was determined by normalization to *β-actin *(equal to 1, dotted line).

The *cldnb:gfp *transgenic zebrafish line expresses membrane-bound GFP in the PLL primordium and neuromasts, in the olfactory system, the ear, branchial arches, and a lower amount of expression in the pronephros and skin in embryos and larvae [1]. As we wished to isolate mostly primordium and neuromast cells -and avoid other labeled cell types- we proceeded to dissect 36 hpf and 48 hpf embryo tails by sectioning the animals at the end of the yolk extension (posterior to the cloaca) (Figure 1). At 36 hpf the PLL primordium has migrated beyond the yolk extension (Figure 1A-B) and, therefore, GFP-labeled cells in the tail sections include almost exclusively lateral line precursors. To confirm proper recovery of the migrating primordium at 36 hpf, isolated tail fragments were observed under fluorescence (Figure 1C). We then processed them for RT-PCR using primers specific for genes with known expression patterns in the PLL such as *cxcr4b *[20], *epcam *[26] and *cldnb *[1] (Figure 1D). Additionally, we carried out *in situ *hybridization to detect expression of *eya1*, a gene specific to the primordium at this stage [27] (data not shown). These controls all showed that, under our experimental conditions, the primordium was present in the entire population of dissected tail fragments. Embryo tails were immediately processed for cell isolation. Cells were passed through a flow cytometer and two cell populations were separated: GFP+ and GFP- (Figure 2A-B). The GFP window selected for flow cytometry indicates that purity was higher than 95%.

To confirm that we were able to achieve enrichment of primordium cells from the 36 hpf fraction by selection on the basis of GFP labeling, we prepared mRNA from both sorted samples of cells and carried out quantitative RT-PCR (qRT-PCR) using primers specific for genes known to be highly expressed in this tissue (*epcam*, *cldnb*) and a muscle-specific gene (*myod*), which should be enriched in the GFP- fraction. As expected, the *epcam *and *cldnb *mRNAs are enriched in the GFP+ population of cells while *myod *is overrepresented in the unlabeled collection of cells (Figure 2C). Similarly, qRT-PCR detection of GFP mRNA showed high enrichment (16 fold) in the GFP+ group of cells. These results indicate that we were able to purify lateral line primordium cells from zebrafish embryo tails at this stage.

### Isolation of neuromast cells (48 hpf set)

A parallel experiment was carried out using 48 hpf *cldnb:gfp *embryos to isolate cells belonging to deposited neuromasts and interneuromastic cells, lying between neuromasts (data not shown). This preparation was carried out in order to have a sample of RNA obtained from the same tissue but at a different developmental stage. Given that these cells are beginning to differentiate and they are no longer migratory, comparison with the primordium sample would allow us to discriminate between genes involved in the different cellular processes occurring during lateral line development. As in the earlier stage, RNA was prepared from GFP+ and GFP- cells obtained from 48 hpf embryos.

### Microarrays and data analysis

#### Identification of the PLL primordium and neuromast transcriptomes

**Figure 3:**
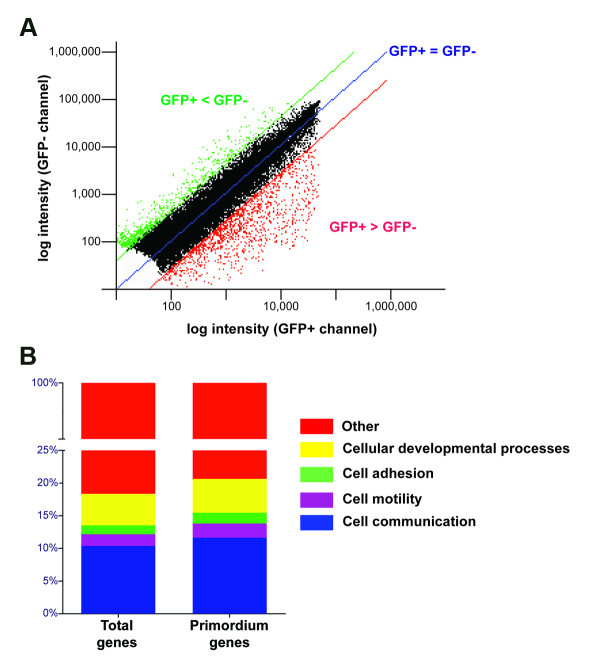
**Transcriptome analysis of zebrafish PLL primordium (36 hpf)**. (A) Scatter plot comparison of gene expression in GFP+ and GFP- in 36 hpf embryos. Each point represents a single transcript/spot on the array, plotted as function of its expression level for GFP+ and GFP-. The blue line represents equivalent expression levels for GFP+ and GFP-. The red line represents a 1.5-fold increased expression level of GFP+ vs. GFP-, whereas the green line represents a 1.5-fold decrease in expression level of GFP+ vs. GFP-. (B) Bar graphs showing the representation of selected molecular functions among the total oligos present in the microarrays and the enriched oligos in the purified GFP+ cells of 36 hpf embryos.

For isolating lateral line-specific transcripts, we reasoned that we could subtract the transcriptome obtained from the sorted GFP- cells to the one obtained from GFP+ cells by carrying out a co-hybridization experiment using microarrays. The sorted GFP- sample contains unlabeled cell types belonging to many different tissues of the same embryos and should thus allow us to remove most housekeeping transcripts from the lateral line transcriptome. We obtained RNA from GFP labeled and unlabeled cells from two independent tail sectioning experiments for each stage analyzed. From each of these samples, cDNA synthesis was carried out and four rounds of dye labeling (including one dye swap) were done for probe generation. "In house" microarray slides printed with 34,000 oligonucleotides that correspond to 19,000 zebrafish-expressed sequences were co-hybridized with probes prepared from GFP+ and GFP- cDNA from each stage analyzed. Differential expression was considered significant when the average hybridization signal from all 4 hybridizations differed by a factor of more than 1.5 (p < 0.05) in either direction (Figure 3A results for the 36 hpf set, 48 hpf not shown).

We have grouped the data obtained from our analysis of differentially detected sequences in a number of tables (Supplementary data). We identified 4,449 (36 hpf set) sequences enriched in the primordium, that represent 3,505 known genes and 944 ESTs that do not map uniquely to a single gene; and 3,425 genes and 575 ESTs (48 hpf set) in the neuromasts (Additional files 1 and 2, respectively). Using the GeneSifter software, our data for the 36 hpf set shows representation of a wide variety of biological functions among the genes identified in this screen (Figure 3B). Gene ontology classification shows that, compared to the relative proportion of cellular functions found in the entire collection of genes represented on the array, some categories are over-represented in primordium cell mRNAs. There is an enrichment in specific biological roles that include, cell motility, adhesion, cell communication and cellular developmental processes. These results are in accordance with known biological processes occurring during migration of the lateral line primordium [8].

Given the diversity of cellular functions involved in development of the lateral line, we combined the results from our two data sets (36 and 48 hpf) and grouped some of the sequences obtained according to known biological processes or developmental pathways (Table 1). Genes implicated, for example, in cancer progression, immune function, cell adhesion and cell signaling were found in this study, many of them described previously in lateral line cells, others being novel. The data provided in this work should help to pinpoint some of the molecular players in lateral line development and provides further parallels with diverse biological functions in normal and pathological situations.

**Table 1:** Upregulated genes classified according to biological process.

BIOLOGICAL PROCESS		Genes differentially expressed detected in this study
Immune system, circulatory	Directed migration	*cxcr4*, *cxcr7*, **similar to hCXCR6+**, *scyba+*; efnb*; ***slit2***; ***sema3+***, ***sema4***

	Innate immunity	*nfkbiaa*; ***irf5***, ***irf7***, ***irf11***; ***ticam1***

	Hematopoiesis	***klf2***, ***klf3+***, *klf4*, ***klf10, klf11***; ***ptgs2+***, ***ptgds+***, ***ptgisl+***

Cell adhesion, epithelial integrity		***cdh1***, ***cdh2, cdh4***, ***cdh***; ***itga6***, ***itgb4***; *cldnb*, cldn7*+, cldnh*, ocln*; *epcam*, ***pecaml+***; f11r*+; cd9*+, ***cd99+***

Morphogenesis		***arx***; ***otx1***; ***otx2***; ***sox9***

Neurogenesis		*atoh1, **atoh2+***; *bdnf*; dlx*; msx*; sox21*

DISEASE		

Cancer	Metastasis	***mmp2, mmp9***, *mmp14*; ***c-myb***, *c-myc*; *stat3*; *c-met*, ***gab1***, ***grb10***, *spint1*; *amotl2*; *st14*; ***prss8, adam9, adam23***

DEVELOPMENTAL PATHWAYS	Wnt related	***wnt4***, ***wnt8***, *wnt10*, ***wnt11***, ***fzd3***, ***fzd4***, *fzd8*, *dkk1+*, ***gsk3b***, *apc*, ***tcf2***, ***gro2***, *vangl2*

	Nodal/TGFβ-related	***ndr1+***, ***bambi***, ***smad4***, ***oep***, ***foxa1***, ***foxa3***, ***sox17+***

	FGF related	*fgf3, **fgf4, fgf8*****, *fgf13***, *fgfr1*

	Hedgehog related	***shh, ptc2***

*described only in ZFIN

+verified by qRT-PCR or in situ hybridization

The table shows a subset of the genes detected in our study that are associated with diverse biological processes. Highlighted in bold are those that have not been shown previously to be expressed in lateral line development. These functions highlight the versatility of the lateral line system for uncovering molecules important for immunity, morphogenesis, neural development and cancer.

#### Validation of the microarray data

In order to estimate the degree of success of our strategy for identifying specifically expressed sequences, we collected information from publicly available sources (ZFIN and PubMed) to identify all genes previously known to be expressed in the lateral line system (primordium and/or neuromasts). We excluded genes expressed only at the placodal stage (PLL precursors), in neural components (PLL ganglion, nerve) and in glial cells. These cell types do not express GFP in the *cldnb:gfp *transgenic line and thus would not have been purified during the cell sorting procedure. In our database survey, we found 372 genes in which specific expression in the PLL primordium or neuromasts has been convincingly described (Additional file 3). We then compiled how many of those genes where actually represented on our chips, we found 267 genes. Out of those, 148 genes where overrepresented in our two data sets (from 36 hpf and 48 hpf embryos) meaning that we were able to detect more than half of the previously described genes and had a 68% success rate if the genes were represented on the chip. Some genes will escape this analysis, particularly genes that have a function in both the migrating primordium and in other tissues of the tail, but the initial capture of genes enriched in the migrating primordia marked by *cldnb *expression appears to be primarily limited by representation on the array.

#### Expression and functional analysis of newly identified genes in the migrating primordium

**Figure 4:**
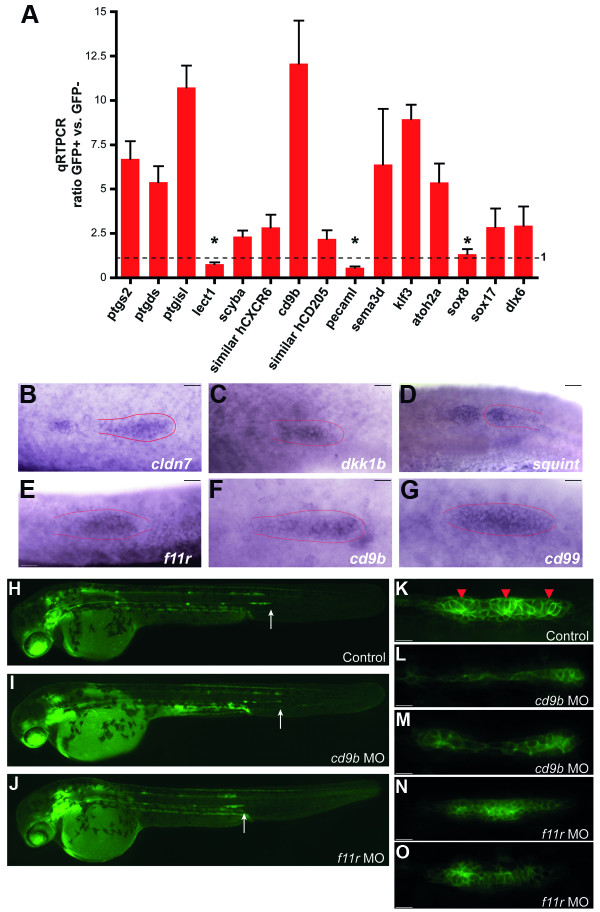
**Validation of microarray analysis**. (A) Quantitative RT-PCR for individual genes with different biological roles was performed of RNA derived from GFP+ and GFP- cells from tails of 36 hpf embryos. Real time PCR ratios were determined by normalization to *β-actin *(equal to 1, dotted line). Only 3 genes out of the 15 tested were not significantly enriched (asterisks). (B-G) *In situ *hybridization of 6 genes enriched in GFP+ cells showing a primordium specific expression pattern in 36 hpf embryos. (H-O) Loss-of-function analysis on two selected genes *(cd9b and f11r) *enriched in GFP+ cells of 36 hpf embryos. *Cldnb:gfp *embryos were injected with anti-sense morpholinos (MO) against *cd9b *(I, L-M) and *f11r *(J, N-O), and compared to control (H, K). White arrows indicate the position where the primordium is at 36 hpf (H-J). Red arrowheads indicate the rosette-like structures in the primordium (K). Scale bars are 10 μm in (B-G) and (K-O).

To provide an independent verification of our results, we randomly selected 15 different cDNAs from sequences showing higher than 1.5-fold enrichment in the GFP+ cells in our 36 hpf set. We performed qRT-PCR to confirm the presence of these transcripts in the purified primordium cells (Figure 4A). For 12 of these genes, quantitative RT-PCR reveled significant enrichment in GFP+ cells.

We wished to provide additional evidence for the success of the purification and selection strategy for identification of genes expressed highly in the lateral line system. We selected differentially expressed genes and the corresponding cDNAs were used as templates for synthesis of digoxigenin-labeled antisense RNA probes for *in situ *hybridization. Specific label was detected in 36 hpf embryos with seven of these probes in the PLL primordium and/or neuromasts (6 of them are shown in Figure 4B-G). The remaining hybridized embryos did not show specific labeling patterns or provided inconclusive results.

Two of the genes, *f11r *and *cd9b*, which displayed enriched expression in the primordium, were selected for loss-of-function analysis, based on their known roles in a large variety of physiological and pathological processes such as the immune response, reproduction and development, infectious and genetic diseases as well as metastasis, where both promote cell adhesion and motility [28-32] and also because of their unknown function in the lateral line development.

F11r (JAM-A) is a transmembrane adhesive glycoprotein that participates in numerous cellular adhesive process including leukocyte migration, intracellular junction assembly and the regulation of cell morphology and motility. In endothelia and epithelia, JAM-A localizes at cell-cell contacts with the tight junction proteins occludin and claudins [28]. In cancer, the tumor progression has been correlated with high JAM-A gene expression levels and patients were significantly more likely to develop metastasis [33]. As tumor cell motility is required early in the metastasis process, is has been speculated that increased JAM-A expression in cancer promote motility, through unregulated β1 integrin protein expression [33]. CD9 in turn, is an integral membrane protein that belongs to the family of tetraspanins and has been implicated in various biological functions, including cell adhesion, motility, metastasis, growth, signal transduction, differentiation, and sperm-egg fusion [31,32].

We inhibited translation of these genes through microinjection of antisense morpholinos (MOs) directed against the ATG region of each transcript. We injected *cldnb:gfp *transgenic zygotes and examined the development of the PLL system from 24 to 48 hpf. Neither of the two MOs elicited gross developmental abnormalities, as was the case for the corresponding control (a nonsense morpholino, not shown). In early LL development, the overall migration process seemed unaffected in either *f11rMO *or in *cd9bMO *injected embryos (Figure 4H-J, white arrows). However, in 50% of *cd9b *morphants (Figure 4L-M) and in 30% of *fr11*MO injected larvae (Figure 4N-O) at 36hpf, we observed differences in the shape and in the internal organization of the primordium compared to control primordia (Figure 4K). Differences were also seen in the neuromast deposition pattern at 48 hpf (Additional file 4). Control embryos show the stereotypical neuromast distribution (Additional file 4: Supplementary Figure 1A) while, in *cd9b *morphants, the neuromast number in the PLL is reduced (Additional file 4: Supplementary Figure 1B-C, arrows). In contrast, in *f11r*MO embryos we observed alterations in the timing and spacing of the deposited neuromasts (Additional file 4: Supplementary Figure 1D-E). These results support previous work [4-6] that the internal organization of the primordium is critical for morphogenesis and patterning of the organ, which is in turn needed for correct neuromast deposition.

#### Pathway analysis

**Figure 5:**
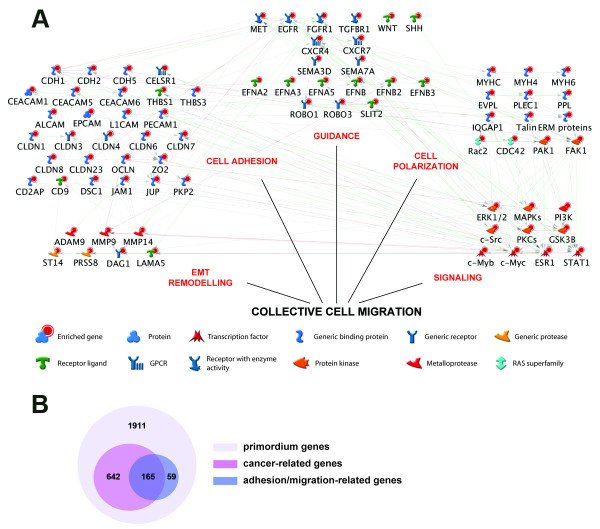
**Pathways analysis on the primordium enriched genes**. (A) Network view of primordium-enriched genes functionally linked by the MetaCore software (GeneGo). Red bubbles show the enriched genes in the purified GFP+ cells of 36 hpf embryos compared to the experimental control. Nodes were connected if functional interactions between the genes are known in their human orthologs. Genes were grouped in the network according to their role in the collective cell migration. (B) Venn diagram showing the interactions of genes enriched in the migrating primordium involve in cell adhesion-migration process and/or cancer metastasis.

In order to determine possible pathways and biological relationships in our 36 hpf data set, we searched for functional interactions among enriched genes using the GeneGo Bioinformatics software. Of the primordium-enriched set of 4,451 zebrafish genes, we identified 1, 911 human orthologs. We focused on the Gene Ontology (GO) terms for the following 5 categories: cell-cell adhesion, guidance, collective cell polarization, EMT remodeling and signaling (Figure 5A). All of these 5 categories have been previously implicated in collective cell migration [8]. As expected, by examining the microarray data from the migrating primordium, we detected an enrichment in genes known to be involved in cell movement, such as *cxcr4*, *cxcr7*, *fgfr*, *egfr *and *matrix metalloproteases *[34]. We also reconstituted the functional network of candidate genes using the known interactions between human orthologs (with GeneGo), which revealed new potential crosstalk between different pathways that are essential for cell migration.

Interestingly, all the enriched genes captured in these categories have demonstrated roles in cancer progression. Furthermore, the many interactions found between these groups of genes, corroborate that collective cell migration results from a complex molecular cascade where dynamic processes occur in a coordinated fashion.

Additionally, we compiled the complete list of genes with known functions in cell adhesion, migration and cancer metastasis provided by GeneGo (Figure 5B) to identify how many of those genes where represented in our dataset. Of the 1,911 primordium-enriched genes (light purple circle in 5B) we founded 807 cancer-related genes (pink circle in 5B) and 224 that are cell adhesion and/or migration-related (blue circle in 5B). Moreover, we found that 165 genes are shared by all events, confirming that cancer progression and normal cell migration in the developing zebrafish involve common mechanisms and pathways.

Thus, we propose that the PLL migrating primordium is a relevant model in which to study the diverse processes that occur during collective cell migration in normal and pathological conditions.

## Discussion

Our aim was to uncover a set of genes expressed in the lateral line primordium and in developing neuromasts, the principal components that form the mechanosensory lateral line system in the zebrafish. We took advantage of the *cldnb:gfp *transgenic line, which expresses GFP in all of the cells derived from the posterior lateral line primordium. GFP positive cells were isolated through cell sorting and the mRNA prepared from these cells was reverse transcribed and probes generated; GFP negative cells were treated similarly to generate probes for subtraction in microarray hybridization experiments. Our reasoning was that this strategy would reveal a collection of genes whose expression was specific to lateral line cells. While this experimental design allowed us to detect genes highly expressed in primordium cells in relation to other tissues -and to discard housekeeping genes-, it was obviously limited by the fact that genes expressed both in the lateral line cells and at the same time in one of the GFP negative cell types, would go undetected. Indeed, as our results show (Additional file 5), not all genes previously described as expressed in the lateral line primordium or neuromasts were recovered in our screen. To date, about 372 genes have been characterized as having high levels of mRNA expression (detectable by *in situ *hybridization) in these organs and thus may have specific roles in development of the lateral line. Our estimates, comparing publicly available data with our results, show that we have detected between half and two thirds of the genes expressed specifically in the lateral line primordium and neuromasts. We speculate that the remaining genes are either expressed simultaneously in other tissues, their expression levels are only slightly elevated in the primordium and neuromasts vs. other cell types, or the appropriate oligonucleotide is absent from the gene array that was used in this study. Despite these caveats, we believe that we have successfully identified a significant fraction of the genes expressed at two different stages of lateral line development: primordium migration and neuromast formation. Moreover, the recovery of genes previously known to be expressed in the LL indicates that the sorting process itself has relatively modest effects on their normal expression profile. Again, this result indicates that the approach followed in this work is able to correctly identify lateral line-specific genes.

Despite the evidence described above, it is also likely that a number of genes identified in our screen are not expressed in the lateral line system and represent false positive identifications. These genes could be expressed in some of the other cell types that also express GFP in the *cldnb:gfp *transgenic line. In the trunk and tail of the transgenic embryos there is detectable expression of the reporter in the pronephros and in skin cells and, given the wide selection window used in the cell sorting procedure and the sensitivity of this technique, there is likely to be contamination of our sorted cell sample with these other cell types. Further exploration of the identified gene collection by *in situ *hybridization will determine the extent of false positive sequences incorporated in our sample. One strategy we followed for increasing the likelihood of identifying lateral line-specific genes was to consider only those overrepresented in both the primordium and neuromast cell selection experiments (36 hpf and 48 hpf; Supplementary Table 4). Additionally, it may be possible to obtain a superior level of enrichment if a transgenic GFP line with higher expression specificity becomes available. Moreover, a different gene identification approach might be warranted, such as deep sequencing of cDNA tags (*i.e*., SAGE) [35], which does not rely on arrayed sequences that are inherently biased.

This study provides a novel set of candidate genes potentially having roles in primordium organization, migration and lateral line differentiation. Formation of the lateral line system is a highly complex and coordinated process in which cell migration, morphogenesis, neural specification and cell differentiation occur simultaneously. Thus, this system offers a unique opportunity to analyze these biological processes in a simple and accessible manner, and reaffirms the conviction that the study of the general principles governing cell migration and fate determination can be studied using the lateral line of the zebrafish. Our study further opens the door for molecular dissection of these processes by increasing the repertoire of genes that are known to be expressed during lateral line development and relate these genes to diverse biological functions (Table 1).

For example, genes implicated in cancer progression and metastatic behavior such as matrix metalloproteases (*e.g*., *mmp9 *and *mmp14*) [36], the ADAM metalloproteinases (*e.g*., *adam9 *and *adam23*) [37] and the tyrosine kinase receptor *c-met*, were detected in this study. *C-met *itself is strongly expressed in the migrating primordium [38] and we also find increased expression of downstream effectors of this pathway such as *gab1*, *shp2, stat3, pi3k, pak1 and Mitogen-activated protein kinases *[39].

Cell adhesion molecules such as *f11r*, *cd9*, *cd99 *and members of the tight junction *Claudin *family genes were also amply represented in our collection. These molecules may be important for maintaining internal tissue organization, coherence of the primordium, pro-neuromast deposition, and/or allowing coordinated multicellular movements [8].

The collective migration of primordium cells has been the focus of intense study and others have noted the parallels of this cellular behavior with that of invasive cancer cells [6,8,26]. In both events, cells undergo an epithelial to mesenchymal transition, acquire migratory behavior, and progress through extracellular matrix to reach a target. Given the cellular and molecular equivalences between both processes (Figure 5), it should be possible to analyze in further detail some of the mechanisms implicated in cancer progression using the migratory lateral line primordium. And most importantly, we envision that it should also be possible to carry out screens for drugs that inhibit this process using zebrafish embryos, a model system used widely for *in vivo *small molecule drug screens [40].

Another biological process that shows molecular parallels with the migrating primordium is immune cell migration. The involvement of chemokines and their receptors in regulating guidance of the primordium cells is well documented. These molecules were first identified in mammals due to their involvement in homing events in the immune system though lymphocytes migrate singly rather than as ensembles of cells. In addition to the chemokine receptors *cxcr4 *and *cxcr7*, we detected *lect1 *(*Leukocyte Cell Derived Chemotaxin 1*), *scyba *and a sequence with homology to human *CXCR6 *(Gene ID: AW232037), suggesting that the high level of complexity required for directional migration of the PLL primordium may involve additional molecular components.

Interestingly, a number of proteins that regulate the innate immune response were detected in our experiment. Downstream components of the transcription factor NFkB, key regulator of innate immune response, were also expressed in the primordium and/or neuromasts such as Inhibitor of *NFkappab *(*nfkbiaa*), Interferon Regulatory Factor 5 (*irf5*), *7 *(*irf7*) and *11 *(*irf11*), and *ticam1 *(toll-like receptor adaptor molecule 1, previously known as *trif*). Likewise, we found members of the prostaglandin family, such as *ptgs1, ptgs2*, *ptgds *and *ptgisl*, that regulate diverse functions of many cell types of the immune system, including T and B lymphocytes and dendritic cells [25]. It is not clear why these genes should be expressed specifically in the lateral line. One possibility is that neuromasts require a highly active innate immune system given their exposure on the body surface, and neuromast cells must respond quickly to biological hazards from the environment. Interestingly, several authors have noted a high concentration of macrophages surrounding the neuromasts, possibly part of the same defense mechanism [41].

In addition, we have detected the expression of members of well known developmental and cellular signaling pathways. Among these is the Wnt pathway, which has been shown to be crucial for primordium organization [9]. While expression of *apc*, *axin2*, *dickopf*, *frizzled 7a*, *lef1 *and *sfrp1a *have been described [6,42,43], we found new components of this pathway in our survey. Importantly, *wnt4b*, a Wnt ligand, could be implicated in segregation of the different compartments of this group of cells. Likewise, Fgf signaling has been shown as a key regulator during lateral line development [9]. The Fgf ligands, *fgf3 *and *fgf10*, guide the tissue compartmentalization in the primordium for proper rosette formation, required for efficient migration [4,5]. Here, we also found new members of the Fgf pathway, such as *fgf4, fgf8 *and *fgf13*, which could be involved in primordium morphogenesis and collective cell movement. Finally, in our survey we found two genes belonging to the Nodal pathway, *squint *and *sox17*, both highly expressed in forerunner cells (FCs) of the zebrafish gastrula [44]. Nodal/TGFβ signaling has a role in the migration of the FCs and in the subsequent rearrangement into rosette-like epithelial structures during embryogenesis. Therefore, these two genes may be involved in the rosette-like organization of cells to form discrete radial clusters.

Notably, the migrating primordium and the deposited proneuromasts contain precursor cells for the diverse cell types that later on differentiate to form a functional sensory organ. Therefore, it is expected that these cells will express neural progenitor cell markers, genes related to neurogenesis, and genes involved in differentiation of the mechanoreceptor hair cells. Most of the known neural progenitor cell markers expressed in the PLL primordium and neuromasts are also expressed in other tissues, including the CNS [19]. Accordingly, these went undetected in our survey and they include *sox2*, *sox3*, *oct4, pou4f2 *and *prox1*. However, we did see overexpression of *shha *and *b*, a set of genes of the *dlx *family and members of the *msx *family, among others.

The analysis of genes expressed by neurons that innervate neuromasts or by glial cells that accompany the nerve and neuromasts were beyond the scope of this study. However, they are integral components of the functioning lateral line and contribute to its development and may provide important cues for its migration and survival. For example, glial cells appear to be responsible for inhibiting neuromast formation from cells lying between neuromasts [45] by producing an inhibitory factor. The sensory neurons located in the PLL ganglion, provide afferent innervation to hair cells and the pioneering growth cone of the lateral line nerve travels together with the primordium as it migrates. The PLL neurons establish somatotopic innervation of hair cells according to their antero-posterior positions through unknown mechanisms. Clearly, there is coordination between the different components and regulatory relationships involving signaling molecules of diverse types must be playing a role in this process. We have found expression of many secreted factors in the primordium and neuromasts, which could play a role in regulation of glial or neuronal behavior. Among these, brain derived neurotrophic factor, *bdnf*, is expressed in neuromasts and may be crucial in maintaining innervation of hair cells [46].

## Conclusions

In this study, we catalogue a variety of biological process and a wide number of signaling pathways involved in lateral line development. Molecules belonging to diverse cellular and developmental processes were uncovered using this approach, demonstrating its utility for the identification of new genes involved in the morphogenesis, migration and cell type specification within the migrating PLL primordium in the zebrafish. Importantly, some of the identified gene products can also open new insights into the understanding of cell migration, which may be crucial to create efficient therapies for a variety of human diseases.

## Methods

### Zebrafish

Fish and embryos (AB and T/AB5 strains) were maintained in our facility according to standard procedures [47]. The *cldnb:gfp *transgenic line was obtained from Darren Gilmour [1]. All embryos were collected by natural spawning, staged according to Kimmel et al. [48] and raised at 28.5°C in E3 medium (5 mM NaCl, 0.17 mM KCl, 0.33 mM CaCl2, 0.33 mM MgSO4, and 0.1% Methylene Blue) in petri dishes, as described [49]. Embryonic and larval ages are expressed in hours post-fertilization (hpf).

### Embryo dissociation and fluorescence activated cell sorting (FACS)

*Cldnb:gfp *embryos were dechorionated with Pronase (P8811, Sigma) and transferred to glass dishes. We used the posterior third of the transgenic embryos in order to avoid recovering GFP-expressing cells in tissues other than the lateral line. Fish were anaesthetized and the tail was transected with a sharp scalpel adjacent to the end of the yolk extension. Tails of embryos were recovered from 36 hpf (for isolating primordium cells) or from 48 hpf (for neuromast cells) embryos, then transferred to Hank's medium containing 0.25% trypsin and 1 mM EDTA and incubated for 15 to 30 min at room temperature during which they were dissociated mechanically by pipetting them up and down every 5 minutes. The digestion was stopped by adding fetal bovine serum to 10% and cell suspensions were then filtered through 40 μm nylon mesh, washed twice with PBS, pelleted by centrifugation at 600Xg for 2 minutes and resuspended in L-15 medium (Sigma) supplemented with 10% fetal bovine serum. FACS of single cell suspensions was performed at room temperature using a FACSAria Sorter (Becton Dickinson) with a Coherent Innova 70 laser at 488 nm and 200 mW power. GFP+ and GFP- cells were sorted directly into Trizol Reagent (Invitrogen) and stored at -80°C.

### RNA isolation and quantitative RT-PCR

Total RNA was isolated from equal numbers of GFP+ and GFP- cells by extraction with Trizol Reagent, according to the manufacturer's instructions. RNA pellets were resuspended in nuclease-free water (Ambion). RNA integrity was confirmed by visualization of the ribosomal RNA on picochips for the Bioanalyzer (Agilent). Approximately 500 ng to 800 ng RNA was linearly amplified by using the Amino Allyl MessageAmp II aRNA Amplification kit (Ambion) with yields ranging from 12 to 30 μg of aRNA. aRNA samples were split and labeled, half with Cy3 mono NHS ester and half with Cy5 mono NHS ester (CyDyes from GE Healthcare; post-labeling reagents from the MessageAmp II kit).

Quantitative PCR was performed using SuperScript™ III Platinum^® ^SYBR^® ^Green One-Step qRT-PCR (Invitrogen) using 20 ng of RNA and 5 μM gene specific-primers in a 25 μL reaction, according to the manufacturer's instructions. PCR primers were as follows: *atoh2a *5'-GCACGAGCTGTTCACCTGCTAAAAC and 5'-GACGGGATCCTCAAAGGGAAGAG

*β-actin *5'-TGGCCCCTAGCACAATGAAG and 5'-

GCCTCCGATCCAGACAGAGTAT;

*cldnb *5'-GAAGGAATTTGGATGAGCTGCGTGG and 5'-CGACAGCATGATTCCCATCAGTCCG;

*cd9b *5'-GTGATTGGAGGAGTCGGCATCG and 5'-CACTCGCGCACATTCACAAACG;

*dlx6 *5'-CCAGCAGACTCAATACCTGGC and 5'-

CCGCCTTGTTTCAACAGCTTC

*egfp *5'-GACCCTGAAGTTCATCTGCACCACC and 5'-GCGGGTCTTGTAGTTGCCGTCGTCC;

*epcam *5'-ATGGCCAGCCATTTGAGGTTGATG and 5'-GCCGTGCAAGAAAGAACAGAACCA.

*klf3 *5'-CCACAGCCAAGAGAAATCGGTC and 5'-GTGTGCGTCTATGGGCTTTCAG;

*lect1 *5'-GAGTGAAAGCTGAGGTGGCGTC and 5'-CTGGCGTCTCATTCGTGTCGC;

*myod *5'-ATGACACACCAAATGCTGACGCAC and 5'-TCTCTGTGGAAATTCGCTCCACGA;

*pecaml *5'-CGTTGGCCGGATGGTTAAATCC and 5'-GCAGCGATGGGAACTTTCACTC;

*ptgs2 *5'-CATGTTTGCTTTCTTCGCCCAGC and 5'-GTGTTGAACCTCCAGCGTCTCTC 3;

*ptgds *5'-CCTGAAGAGTGATGGTTCCTGCTG and 5'-GTCAACCACGCGCATGTCATTG;

*ptgisl *5'-GAACAACCTCCGCCTGCTTATG and 5'-GCTTGTCAAACCGGCGAAACTC;

*scyba *5'-GATGAATCGCTGTAGTACGGCCGC and 5'-CCGGATCTTCGGCCCTTTTCTTG;

*sema3 d *5'-CAGGTGTCACTGCACAGGTGCC and 5'-GTGTCCCAACAATGGCTGGATG;

*similar hcd205 *5'-GTGCGTCAGATCTCAGTAGTTTGA G and 5'-GCTAAAGCAGTGCACCGAGTGCTG;

*similar hcxcr6 *5'-GAAGAATGGCTGGAACCTGCAAC and 5'-CCGACTATGCCAGTCACAACC;

*sox8 *5'-GAACGCGTTTATGGTTTGGGCTC and 5'-CCTCCACGAACGGCCTCTTCTC;

*sox17 *5'-CCCAGACCTGCACAATGCGG and 5'-GGGCTCCAATCGCTTGTTTCG

Expression levels were determined by comparison to a standard curve from total RNA isolated from whole embryos. Values from GFP- and GFP+ samples were then normalized to *β-actin *to obtain relative expression levels. Standard deviations were calculated from triplicate measurements.

### Microarrays analysis

We used "in house" 34 K zebrafish oligo microarray chips, which was derived from three sources: Compugen (16,512 × 60 mers), MWG (14,240 × 50 mers) and Operon (3,479 × 70 mers). The whole set contains replicates of several positive (known housekeeping genes) and negative control oligos (random sequences) to control for the homogeneity and specificity of the hybridization.

Two biological replicates of GFP+ and GFP- cells were cohybridized, each with one dye swap, for a total of eight hybridizations. Hybridizations were performed overnight at 45°C in Maui Mixer FL hybridization chambers (BioMicro Systems). Microarray slide post-hybridization processing and scanning were done as previously described [50]. Data analysis to identify genes enriched in GFP+ vs. GFP- cells in pair-wise comparisons was carried out with FileMaker Pro 9 (FileMaker, Inc.) and GeneSifter http://www.genesifter.net/ software. Data points with average quality values below 0.7 were eliminated and the datasets were Lowess normalized. Normalized data were log-transformed and GFP+ and GFP- values were separately averaged over the eight experiments. Fold differences were calculated from log averages and Student's t-test with Benjamini and Hochberg correction to generate p-values that were used to determined statistical significance. The microarray data is available at NCBI's Gene Expression Omnibus and are accessible through GEO Series accession number GSE25617.

### In situ hybridization

Whole mount *in situ *hybridizations (ISH) were carried out essentially as described in [51], with post-hybridization washes performed by an automated liquid exchanger (Biolane HTI, Hölle & Hüttner). Plasmids containing zebrafish cDNAs and EST clones were obtained from OpenBiosystems (Huntsville, AL). Inserts from plasmids were PCR amplified using specific primers containing RNA polymerase binding sites and purified using Qiaquick columns (Qiagen). Antisense digoxigenin-labeled riboprobes were synthesized from PCR products using the appropriate bacteriophage RNA polymerase.

### Pathway analysis

Pathway analysis of the genes enriched in the primordium was done with MetaCore software (GeneGo). The known relationships between the human orthologs of the zebrafish genes were used to generate the interaction network. The network was then manually examined and curated. In addition, an extensive search of the "GeneGo processes" was done for genes known to be involved in cell migration/adhesion and cancer pathways. The primordium-enriched genes involved in two or more of these processes were identified.

## Competing interests

The authors declare that they have no competing interests.

## Authors' contributions

VEG designed and performed all the experiments. JL participated in the microarray and pathways analysis. AE carry out the microarray experiments. MB, EJV and VR participated in the design of the study and established the cell sorting conditions. MLA and SMB designed the experiments, contributed to the conclusions and drafted the manuscript. All authors read and approved the final manuscript.

## Supplementary Material

Additional file 1 **Supplementary Table 1**. Complete list of genes enriched in microarray experiment at 36 hpf. Click here for file

Additional file 2 **Supplementary Table 2**. Complete list of genes enriched in microarray experiment at 48 hpf. Click here for file

Additional file 3 **Supplementary Table 3**. Known PLL primordium and neuromasts markers. Click here for file

Additional file 4 **Supplementary Figure 1**. Pattern of the neuromast deposition in the embryonic PLL. The number and position of the neuromasts was analyzed in control (A), *cd9b*MO (B-C), and *f11r*MO (D-E) embryos at 48 hpf. Red arrows indicate the position of the PLL neuromasts. Click here for file

Additional file 5 **Supplementary Table 4**. List of genes enriched at 36 and 48 hpf. Click here for file
