# Supplementary figures and images for: Molecular dissection of the migrating posterior lateral line primordium during early development in zebrafish
Source: BMC Dev Biol. 2010 Dec 13;10:120. doi: 10.1186/1471-213X-10-120 (PMC3016277; doi:10.1186/1471-213X-10-120)

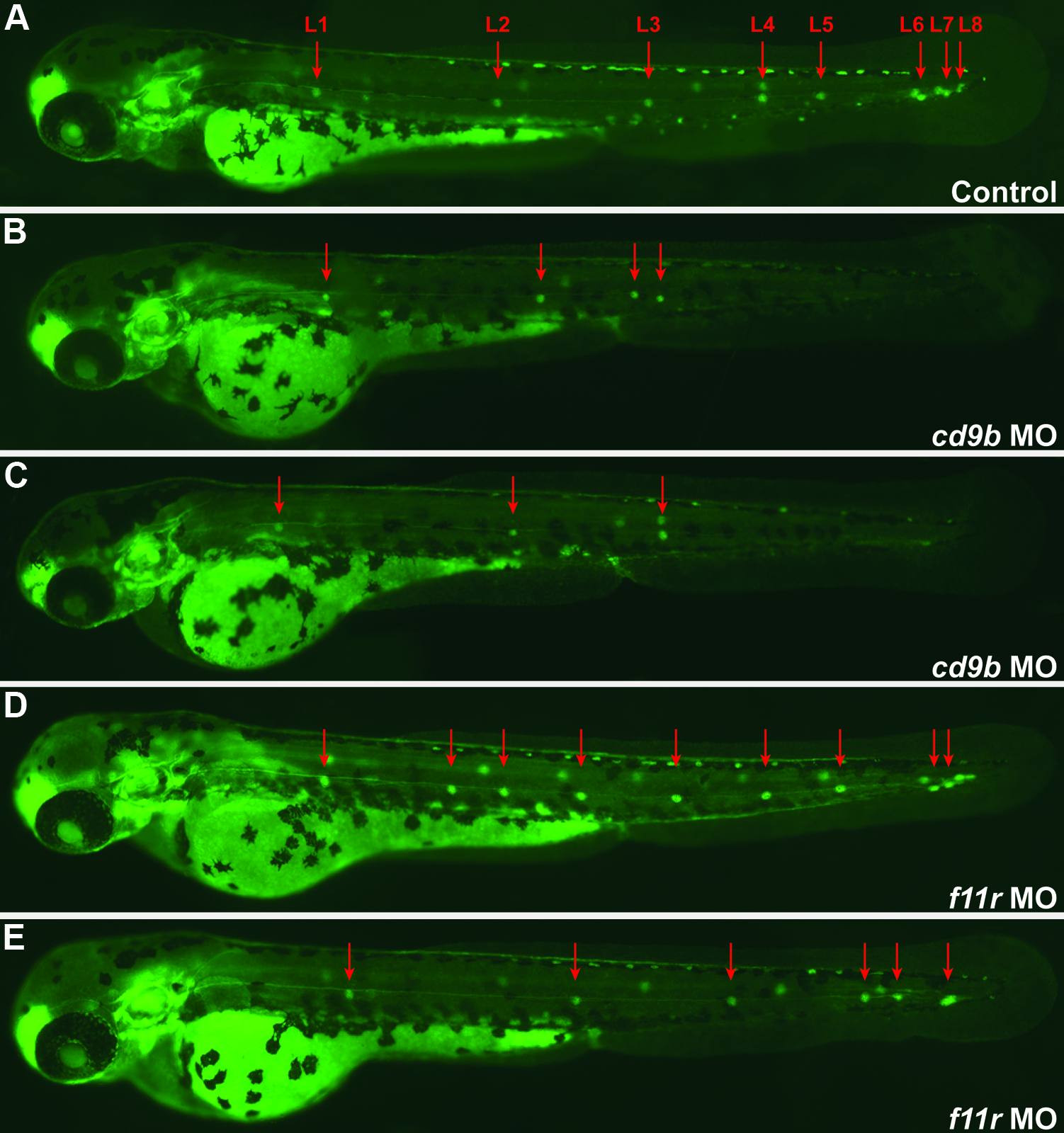

Supplement: Additional file 4 — Supplementary Figure 1. Pattern of the neuromast deposition in the embryonic PLL. The number and position of the neuromasts was analyzed in control (A), cd9bMO (B-C), and f11rMO (D-E) embryos at 48 hpf. Red arrows indicate the position of the PLL neuromasts. [file 1471-213X-10-120-S4.TIFF]
